# The 5 Alpha-Reductase Isozyme Family: A Review of Basic Biology and Their Role in Human Diseases

**DOI:** 10.1155/2012/530121

**Published:** 2011-12-25

**Authors:** Faris Azzouni, Alejandro Godoy, Yun Li, James Mohler

**Affiliations:** Department of Urology, Roswell Park Cancer Institute, Elm and Carlton Streets, Buffalo, NY 14263, USA

## Abstract

Despite the discovery of 5 alpha-reduction as an enzymatic step in steroid metabolism in 1951, and the discovery that dihydrotestosterone is more potent than testosterone in 1968, the significance of 5 alpha-reduced steroids in human diseases was not appreciated until the discovery of 5 alpha-reductase type 2 deficiency in 1974. Affected males are born with ambiguous external genitalia, despite normal internal genitalia. The prostate is hypoplastic, nonpalpable on rectal examination and approximately 1/10th the size of age-matched normal glands. Benign prostate hyperplasia or prostate cancer does not develop in these patients. At puberty, the external genitalia virilize partially, however, secondary sexual hair remains sparse and male pattern baldness and acne develop rarely. Several compounds have been developed to inhibit the 5 alpha-reductase isozymes and they play an important role in the prevention and treatment of many common diseases. This review describes the basic biochemical properties, functions, tissue distribution, chromosomal location, and clinical significance of the 5 alpha-reductase isozyme family.

## 1. Introduction

Testosterone (T) is the most abundant androgen in serum. Approximately 97% of T is bound to albumen and sex-hormone binding globulin and the remaining 3% is free and biologically active. T is synthesized by the Leydig cells of the testes under the control of the hypothalamus and anterior pituitary gland. In male fetuses, T stimulates the differentiation of the Wolffian duct into male internal genitalia (epididymis, vas deferens, and seminal vesicles) and development of libido, enlargement of the vocal cords, skeletal muscles, penis, and scrotum and the initiation of spermatogenesis at puberty [1, 2]. T is taken from circulation to cells through processes that remain poorly understood. Intracellular T is converted to dihydrotestosterone (DHT), the preferred ligand for androgen receptor (AR) transactivation, by the enzyme 5 alpha-reductase (5*α*-R). Upon ligand binding and transactivation, the DHT-AR complex translocates from cytoplasm to nucleus and activates the transcription of certain genes (the androgen receptor-regulated genes, ARRG).

DHT is important for *in utero* differentiation and growth of the prostate gland, male external genitalia (penis and scrotum), and pubertal growth of facial and body hair. DHT plays an important role in several human diseases, which include acne, hirsutism, male pattern baldness, benign prostate hyperplasia (BPH), and prostate cancer (CaP) [3]. The role of DHT was discovered after the description of 5*α*-R2 deficiency in a group of males from the Dominican Republic [4]. DHT has 2–5 times higher binding affinity for AR than T, and 10-fold higher potency of inducing AR signaling than T [5], which means that their effects are different but complementary [6].

Three isozymes of 5*α*-R are known to exist (5*α*-R1-3) [7] and two other proteins exhibit 5-alpha reducing capabilities, glycoprotein synaptic 2 (GPSN2), and glycoprotein synaptic 2-like (GPSN2L) proteins. Only one 5 beta-reductase (5*β*-R) enzyme has been identified. Its products, 5*β*-isomers, are labeled as epi-product, such as 5*β*-DHT (epi-DHT) [8]. Several compounds have been developed to inhibit the 5*α*-R enzyme system and they play an important role in the prevention and treatment of many common diseases [9]. This review describes the basic biochemical properties, functions, tissue distribution, chromosomal location, and clinical significance of this enzyme family.

## 2. Background

**Figure 1:**
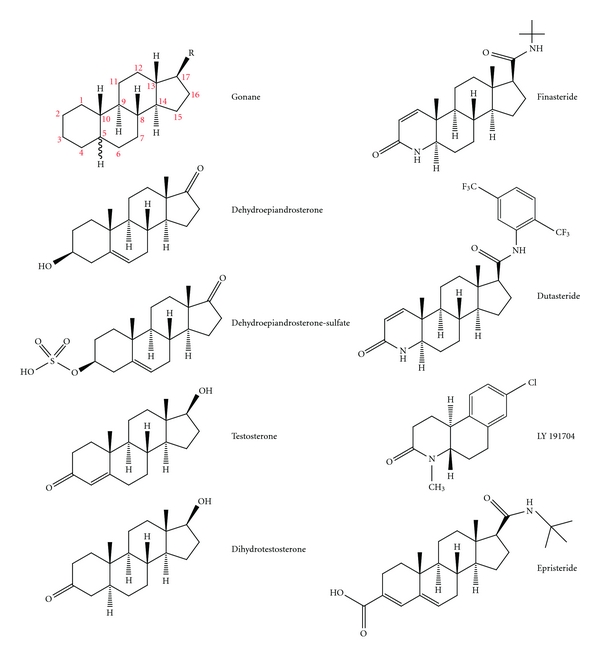
Structure of various steroids.

Steroids are a special type of lipid. The backbone of steroids is the compound “gonane”, a 17-carbon molecule composed of 4 rings. The three cyclohexane rings are labeled A, B, and C. These 3 rings together are called phenanthrene. Ring D is a cyclopentane ring. The carbon atoms are numbered from 1 to 17. Typically, steroids have a methyl group (–CH_3_) at carbons C-10 and C-13 and an alkyl side chain (R) at C-17 (Table 1). Alkanes are saturated hydrocarbons composed of carbon and hydrogen atoms linked by single bonds. The simplest alkyl group is a methyl group. Steroids vary by the configuration of the alkyl side chain, the number of additional methyl groups, and the functional groups attached to the steroid nucleus. Carbons number 18 and 19 are attached to carbons number 13 and 10, respectively. Additional carbon atoms are usually a part of the R side chain or attached elsewhere to the steroid backbone [22]. Androgens are derivatives of androstane and contain 19 carbons and either a keto group (e.g., dehydroepiandrosterone (DHEA) and androstenedione (ASD)) or a hydroxy group (e.g., T and DHT) at position 17 of the steroid nucleus (Figure 1).

## 3. Historical Overview

Steroid-5-reductases (5*α*-R and 5*β*-R) were first discovered, purified, and characterized in rat liver homogenates [23]. These early experiments demonstrated that these enzymes were capable of irreversibly reducing the delta 4, 5 bond (double bond between carbons 4 and 5; Δ^4,5^) of C-19 and C-21 steroids to 5*α*- and 5*β*-stereoisomers.

The first androgen isolated was androsterone, a 5*α*-reduced androstane, which was isolated by Butenant in 1931 from 25,000 liters of urine from adult men. This steroid was assumed to be the male hormone until 1935 when Ernst Laquer and his colleagues isolated T from several tons of bull testes. The 5*α*-R enzyme was characterized initially in the 1950s in rat liver slices based on its ability to convert deoxycorticosterone to 5*α*-reduced metabolites [24]. Tomkins and others showed that the enzyme required a reduced pyridine nucleotide cofactor (i.e., NADPH) and could metabolize a variety of steroid substrates [25]. Speculation persisted about whether a single enzyme or multiple enzymes were involved in 5*α*-reduction of steroids. The 5*α*-reduction of steroids made them susceptible to further reduction, sulfation, and glucuronidation, modifications that decreased their affinity to bind proteins, made them more hydrophilic and facilitated their excretion. In the 1960s, 5*α*-reduction was shown to be an irreversible reaction and DHT was found to be a more potent androgen than T in prostate bioassays [26]. The administration of radiolabeled T to rats resulted in a time-dependent accumulation of DHT in the nuclei of ventral prostate cells, which subsequently bound to a specific nuclear (androgen) receptor. These data indicated that 5*α*-reduction of T is a crucial step in androgen action and focused attention on 5*α*-R. The central role of 5*α*-R in mammalian male physiology was obtained from developmental studies of mammalian embryos showing that 5*α*-reduction activity was highest in the primordia of the prostate and external genitalia prior to their virilization, but very low in Wolffian duct structures [27, 28], and from genetic studies on a rare disorder of male sexual differentiation, originally termed pseudovaginal perineoscrotal hypospadias and subsequently referred to as 5*α*-R deficiency [4]. Analysis of enzyme activity in skin samples and of urinary and serum steroids revealed a generalized defect in the conversion of T to DHT.

Studying 5*α*-R was hampered by the insolubility of the protein, a hurdle which was overcome in 1989. The technique of expression cloning in Xenopus laevis oocytes was used to isolate a cDNA from rat liver that encoded 5*α*-R enzyme, which was used to isolate a human 5*α*-R by cross-hybridization with a prostate cDNA library. The two expressed proteins had different biochemical properties and different responses to finasteride. These observations suggested the presence of two 5*α*-R isozymes that were confirmed by studies done in patients with 5*α*-R deficiency. The coding sequence of the gene specifying the rat liver cDNA was isolated and found to be normal in these patients. Further genetic studies in these patients identified a different mutated gene that encoded a 5*α*-R in normal individuals with identical biochemical properties to the human prostatic 5*α*-R. The first cDNA, isolated from rat liver, was named 5*α*-R1 (SRD5A1) gene, and the second cDNA, which was isolated from human prostate and found defective in 5*α*-R-deficient patients, was named 5*α*-R2 (SRD5A2) gene [29]. 

More recently, with the development of genome-wide gene expression profile analyses, a third 5*α*-R (SRD5A3) gene was identified. GPSN2 and GPSN2L proteins were identified using sequence searching and NCBI's BLAST (http://blast.ncbi.nlm.nih.gov/Blast.cgi). All primary species (from plant, amoeba, yeast, to vertebrate) in Eukaryota contain all 3 subfamilies [8]. 

## 4. Family Members

The 5*α*-R family is composed of 3 subfamilies and 5 members (isozymes) in total. Isozymes are different proteins that perform the same function:

- 5*α*-R1 and 5*α*-R2,
- 5*α*-R3,
- GPSN2 and GPSN2L proteins.

## 5. Functions

### 5.1. 5 Alpha Reduction: (5*α*-R1-3) [29, 30]

The substrates for 5*α*-reductases are 3-oxo (3-keto), Δ^4,5^ C 19/C21 steroids. The group “3-keto” refers to the oxygen-carbon double bond at carbon 3. Delta 4, 5 refers to the double bond between carbon atoms 4 and 5. The reaction involves a stereospecific, irreversible breakage of the double bond between carbons 4 and 5 (delta 4, 5) with the aid of cofactor NADPH and the insertion of a hydride anion (H^−^) to the *α* face at carbon C-5 and a proton to the *β* face at position C-4. Examples of substrates are T, progesterone, androstenedione, epi-T, cortisol, aldosterone, and deoxycorticosterone. The physiologic role of 5*α*-reduction of these steroids (other than T) is unknown but probably related to their degradation and excretion or to certain physiologic functions. 5*α*-dihydroprogesterone (5*α*-DHP) is a major hormone in the circulation of both normal cycling and pregnant women [31]. 5*α*-dihydrocortisol is present in the aqueous humor of the eye, is synthesized in the lens of the eye, and may play a role in the regulation of aqueous humor formation [32]. 5*α*-Dihydroaldosterone is a potent antinatriuretic agent with somewhat different physiologic effects than aldosterone itself; its formation in the kidney is enhanced by restriction of dietary sodium intake, which suggests its importance for the conservation of sodium [33]:

(1) $$\begin{array}{c} \left(\text{Substrate}\right)+\left(\text{NADPH}\right)+\left({\text{H}}^{+}\right)\;\\ \;\;\to \left(5\alpha \text{-substrate}\right)+\left({\text{NADP}}^{+}\right) \end{array}$$

Uemura et al. used small interfering RNA (siRNA) to knock down the expression of 5*α*-R3 isozyme in 22RV1 and LNCaP-C4-2 CaP cells by transfecting them with several siRNA expression vectors [34]. Subsequently, they studied mRNA expression of 5*α*-R3 (RT-PCR), cell growth and viability, and the ratio of DHT/T using liquid chromatography-tandem mass spectrometry. Knockdown of 5*α*-R3 expression caused decreased cell growth and viability and DHT/T ratio. Unpublished work from our group has confirmed the ability of 5*α*-R3 to 5*α*-reduce 3-oxo, delta 4,5 C19 and C21 (T, androstenedione and progesterone) steroids in lysates of CHO-K1 cells transfected with 5*α*-R3 cDNA via an adenovirus vector, CaP cell lines CWR-22 and CWR-22R, and clinical human samples of androgen-stimulated benign prostate (AS-BP), androgen-stimulated (AS-CaP), and castration-recurrent (CR-CaP) CaP.

### 5.2. N-Glycosylation of Proteins: (5*α*-R3)

Congenital deficiency of 5*α*-R3 has been linked to a rare, autosomal recessive disorder in which patients are born with mental retardation, cerebellar, and ophthalmologic defects [35]. The presumed defect involves the reduction of the terminal double bond of polyprenols to dolichols, an important step in protein N-glycosylation. N-linked protein glycosylation involves the addition of a 14-sugar glycan to select asparagine residues on a nascent protein to facilitate proper folding and trafficking of the protein and occurs in the membranes of endoplasmic reticula. This disorder is part of the family of congenital disorders of glycosylation and was described for the first time in a family in the United Arab Emirates by Cantagrel et al. [35].

### 5.3. Potential Biomarker of Malignancy: (5*α*-R1-3) [14, 20]

The bulk of published literature indicates that the expression of 5*α*-R1 increases and 5*α*-R2 decreases in CaP compared to benign prostate and BPH. Umera et al. confirmed for the first time increased expression of 5*α*-R3 at the mRNA level in CR-CaP. Godoy et al., confirmed this at the protein level. A validated monoclonal antibody showed that expression of 5*α*-R3 was increased similarly in AS-CaP and CR-CaP compared to AS-BP. 5*α*-R3 expression was increased in lung, breast, papillary thyroid, and testicular (seminoma and yolk sac) cancers compared to their benign counterparts.

### 5.4. Erythropoiesis [36]

5*α*-C 19 steroids increase the production of erythropoietin hormone in the kidneys. 5*β*-C 19 steroids are important for heme synthesis in the liver.

### 5.5. Regulation of Bile Synthesis [37]

Both 5*α*-R and 5*β*-R are involved in bile biosynthesis, where they catalyze the conversion of 7*α*, 12*α*-dihydroxy-4-cholesten-3-one into 7*α*,12*α*-dihydroxy- **5*α***-cholestan-3-one, and 7*α*,12*α*-dihydroxy-**5*β***-cholestan-3-one, respectively. Only the 5*β*-isomer has been shown to be biologically active and is used for bile synthesis. The 5*α*-isomer is inactive and suggested to be an inhibitory step in bile biosynthesis regulation in humans.

### 5.6. GPSN2 Family [38]

While the functions of the GPSN2 subfamily are not understood fully, several reports have shown that GPSN2 members are involved in the fourth reaction of fatty acid elongation by reducing a fatty chain double bond in mammals. Although the substrate (fatty acid) of GPSN2 members is structurally different from that of the other two 5*α*-R subfamilies, all three subfamilies of 5*α*-R share a similar biochemical ability of reducing a double bond of the substrate.

## 6. Protein Structure and Gene Location [8, 29, 39]

5*α*-R1 and 2 isozymes are NADPH-dependent, membrane-associated (microsomal) enzymes, composed of 259 and 254 amino acids, and have molecular weights of 29.5 and 28.4 kilodaltons, respectively. They contain a high content of hydrophobic amino acids distributed throughout their sequences, which suggests that they are intrinsic membrane proteins deeply embedded in the lipid bilayer.

Even though these two isozymes are intrinsic membrane proteins and catalyze the same reaction, they only share a limited degree of homology in protein sequence, are located on different chromosomes, and possess distinctive biochemical properties. The average sequence identity between these two isozymes within a given species is approximately 47%, while the sequence identity between the same isozyme across species is 60% for 5*α*-R1 and 77% for 5*α*-R2. They are encoded by the 5*α*-R1 and 5*α*-R2 genes. These genes have similar structures, with five coding exons separated by four introns. The positions of the introns are essentially identical in the two genes. However, SRD5A1 is located on chromosome 5p15 whereas SRD5A2 is on 2p23. Gene polymorphisms exist for the two genes and are more common for 5*α*-R2. More than 850 and more than 550 single nucleotide polymorphisms (SNPs) have been reported for 5*α*-R2 and 5*α*-R1 genes, respectively [40, 41]. Only a few of these gene polymorphisms affect enzyme activity; some decrease (e.g., V89L SRD5A2 variant) and others increase (e.g., A49T SRD5A2 variant) enzyme activity [42]. Molecular epidemiologic studies are inconclusive as to whether altered 5*α*-R2 isozyme activity due to 5*α*-R2 gene polymorphism affects CaP risk [43]. A variant of 5*α*-R1 gene was reported to increase risk of polycystic ovary syndrome (PCOS) and more severe hirsutism in lean women, whereas a variant of 5*α*-R2 gene was associated with decreased risk of PCOS in the same cohort [44]. More than 300 SNPs have been reported for 5*α*-R3 gene; however, their clinical significance remains uncertain [45]. 5*α*-R3 is composed of 318 amino acids and has only 19% homology with 5*α*-R1 and 20% homology with 5*α*-R2 [39]. 5*α*-R3 is encoded by SRD5A3, which is located at 4q12. The genes encoding GPSN2, GPSN2-like, and 5*β*-R are located at 19p13.12, 4q13.1, and 7q34, respectively. GPSN2 and GPSN2-like proteins are composed of 308 and 363 amino acids, respectively. The amino acid sequence homology for GPSN2 is 15% with 5*α*-R1, 17% with 5*α*-R2 and 11% with 5*α*-R3. GPSN2-like has 6%, 11%, 6%, and 44% sequence homology with 5*α*-R1, 5*α*-R2, 5*α*-R3, and GPSN2, respectively.

## 7. Biochemical Properties [8, 29]

When examined in lysates of transfected cells, 5*α*-R1 exhibits a broad pH optimum, which ranges between 6.0 and 8.5, while 5*α*-R2 shows a narrow acidic pH optimum (pH 5–5.5). However, there is evidence to suggest that inside intact human cells, 5*α*-R2 isozyme functions optimally at a more neutral pH range (6.0–7.0). 5*α*-R1 has a larger turnover number, as indicated by its *K*_cat_ value and a lower substrate affinity for T, *K*_*m*_ = 1–5 *μ*M. 5*α*-R2 has a lower turnover number (*K*_cat_) and a higher substrate affinity, as indicated by *K*_*m*_ = 0.004–1 *μ*M for T. Under optimal conditions, 5*α*-R2 has a higher 5*α*-reducing activity than 5*α*-R1, as indicated by its high *V*_max⁡_/*k*_*m*_ ratio. Both isozymes contain an NH_2_-terminal steroid (ligand) binding domain and a COOH-terminal NADPH binding domain. The apparent dissociation constant for NADPH cofactor is similar for both isozymes (3–10 *μ*M). No such comparisons exist for 5*α*-R3 except that it appears to be efficient at pH 6.5–6.9 (unpublished work from our group).

## 8. 5***α***-Reductase Inhibitors [9, 30, 46]

Goal of development of 5*α*-reductase inhibitors (5*α*-RI) was to bind to 5*α*-R with little or no affinity for the androgen or other steroid receptors. The first inhibitors were steroids that mimicked T and, in many cases, were substrates themselves (i.e., not true inhibitors). The inhibitors can be broadly classified into two categories: steroidal and nonsteroidal. The steroidal class has more inhibitors thus far.

The mechanism of 5*α*-RI is complex but involves the binding of NADPH to the enzyme followed by the substrate. The Δ^4,5^ bond is broken and a hydride anion is transferred from NADPH directly to the C-5 carbon on the *α* face followed by a proton attacking the C-4 carbon on the *β* face leading to the formation of the product that subsequently leaves the enzyme-NADP^+^ complex. NADP^+^ departs last and the enzyme becomes free for further catalysis cycles. Based on this, the mechanism of inhibition of 5*α*-R isozymes is divided into three types [30]:

- competitive with the cofactor (NADPH) and substrate (bi-substrate inhibitors): the inhibitor binds the free enzyme, for example, ONO-3805;
- competitive with the substrate: the inhibitor binds the enzyme-NADPH complex for example, 4-, 6-, and 10-azasteroids;
- uncompetitive with the enzyme-NADP^+^ complex: the inhibitor binds the enzyme-NADP^+^ complex after the product leaves, for example, epristeride.

### 8.1. Steroidal 5*α*-RI (Figure 1)

(1) 4-Azasteroids: the 3-oxo, 5-alpha steroids with a nitrogen atom at position 4 have been the most extensively studied. Examples include finasteride (MK-906), dutasteride (GG745), 4-MA, turosteride, MK-386, MK-434, and MK-963.

- Finasteride is a synthetic 4-azasteroid and is the first 5*α*-RI approved for treatment of benign prostatic enlargement (BPE) and subsequently male pattern baldness. Finasteride is a potent (mean inhibitory concentration [IC_50_], 69 nM) competitive inhibitor of 5*α*-R2 but inhibits less effectively 5*α*-R1 (IC_50_ 360 nM) [47]. Finasteride decreases mean serum level of DHT by 71% after 24 weeks of use [48]. Seven-day treatment with finasteride (1 or 5 mg daily) has been reported to suppress intraprostatic DHT in men with lower urinary tract symptoms (LUTSs) attributed to BPE by approximately 85% relative to placebo [49], whereas another study of finasteride 5 mg/d (also in men with LUTS attributed to BPE) demonstrated a reduction of 68% at 6 months [49]. Finasteride was shown *in vitro* to inhibit 5*α*-R3 at a similar potency to 5*α*-R2 (IC_50_ = 17.4 nM, 14.3 nM, resp.) in transfected HEK-293 cells [21].
- Dutasteride is a synthetic 4-azasteroid with a half-life of nearly 5 weeks and is only approved for treatment of BPH. Dutasteride is a dual 5*α*-RI since it is more effective (more potent) at inhibiting 5*α*-R1 and 2 than finasteride; IC_50_ for inhibiting 5*α*-R1 is 7 nM and 5*α*-R2 is 6 nM. Dutasteride reduced mean levels of serum DHT at 24 weeks better than finasteride (94.7% versus 70.8% suppression) [50] and caused a 97% reduction in intraprostatic DHT levels in men with CaP treated with 5 mg/d for 6–10 weeks [51]. Another trial of dutasteride 3.5 mg/d for 4 months prior to RP decreased intraprostatic DHT by 99% [52]. The near-maximal suppression of intraprostatic DHT with dutasteride 3.5–5 mg daily and the report that dutasteride inhibits 5*α*-R3 *in vitro* (IC_50_ = 0.33 nM) [21] suggest that the development of a triple 5*α*-R inhibitor may not be necessary.  Table 2 provides a comparison between finasteride and dutasteride.
- 4-MA was a potent dual inhibitor of 5*α*-R1 (IC_50_ = 1.7 nM) and 5*α*-R2 (IC_50_ = 1.9 nM). 4-MA had a very low affinity for AR and thus was not expected to produce undesirable antiandrogen effects, such as impotence, impaired muscle growth, or gynecomastia. However, 4-MA was withdrawn from clinical development after it was shown to be an inhibitor of 3*β*-hydroxysteroid dehydrogenase and to cause hepatotoxicity [9].
- Turosteride, MK-434, and MK-963 inhibit mainly 5*α*-R2. MK-386 is a selective 5*α*-R1 inhibitor [46].

(2) 6-Azasteroids (e.g., GIlS7669X) have a heterocyclic B ring (nitrogen atom at position 6) and a Δ^4,5^ bond in the A ring and are potent competitive inhibitors of 5*α*-R1 and 2 [46].

(3) 10-azasteroids, for example, AS97004, are competitive 5*α*-RI with a similar mechanism of action to 6-azasteroids [9].

(4) Androstanecarboxylic acids, such as epristeride, are noncompetitive, specific inhibitors for 5*α*-R2 [30].

(5) Other steroidal inhibitors include progesterone esters such as 4-bromo-17*α*-(*p*-fluorobenzoyloxy)-4-pregnene-3, 20-dione [53], 2-azasteroids, 3-azasteroids, 19-nor-10-azasteroids, and diazasteroids [9].

### 8.2. Nonsteroidal Inhibitors [9, 30, 46]

Several pharmaceutical and academic groups have pursued the synthesis of nonsteroidal compounds that inhibit human 5*α*-reductases due to the undesired hormonal side effects of steroidal compounds. Nonsteroidal inhibitors can be classified according to their structure. Most have been derived from azasteroidal inhibitors by removing one or more rings from the azasteroidal structure. Nonsteroidal inhibitors are thought to act as competitive inhibitors with exception of epristeride analogues, which are noncompetitive inhibitors. The most potent and selective inhibitors of human 5*α*-R1 are found among these classes of compounds and include the following:

1. Benzoquinolines include many subgroups.
  - Benzo[f]quinolinones are tricyclic compounds that are derived from 4-azasteroids by removing the D ring and substituting the C ring with an aromatic one. These are selective against 5*α*-R1. The potency against 5*α*-R1 increases by substituting a halogen atom at position 8 (F, Br, or specially a Cl) and a methyl group at position 4. LY 191704 is the most potent (IC_50_ = 8 nM).
  - Piperidones lack B and D rings.
  - Quinolinones lack C ring.
  - Pyridines lack B and C rings.
  - Benzo[c]quinolinones tricyclic compounds derived from 6-azasteroids (no D ring, aromatic ring for the C ring) that have selective but weak inhibitory activity against 5*α*-R1.
  - Benzo[c]quinolizinones are tricyclic compounds derived from 10-azasteroids (no D ring, aromatic ring for the C ring) that include some very potent, selective inhibitors of 5*α*-R1.

(Subgroups (b), (c), and (d)) are very weak 5*α*-R1I.
(2) Nonsteroidal aryl acids are tricyclic compounds derived from androstanecarboxylic acids that differ from their parent compounds in being selective, noncompetitive 5*α*-R1I.
(3) Butanoic acid derivatives contain an aromatic ring (generally benzene or indole) that bears a butanoic acid chain and aromatic moieties. Examples include ONO-3805, demonstrated *in vitro* to be a selective inhibitor of 5*α*-R1, and FK143, which inhibits 5*α*-R1 and 5*α*-R2 equally and noncompetitively.
(4) Polyunsaturated fatty acids, found in vegetable oils, have been found to inhibit human and rat microsomal 5*α*-R activity. In this group, y-linolenic acid is the most potent compound tested. Since 5*α*-R isozymes are intrinsic membrane proteins, their activity may depend on the unique environment of the lipid bilayer. Whether and how fatty acids may function as endogenous regulators of 5*α*-R remain unknown.
(5) Some cations, especially zinc, have been reported to reduce sebum production *in vivo* and have been used to treat acne. *In vitro* assays have indicated that zinc specifically inhibits 5*α*-R1. This inhibition may be mediated both by non-competitive inhibition of T binding to 5*α*-R and by reduced formation of the NADPH co-factor.
(6) Other nonsteroidal inhibitors include epicatechin-3-gallate and epigallocatechin-3-gallate, which are major constituents of green tea. Also included are 7-hydroxycoumarin derivatives, 2,6-disubstituted 4-hydroxy-4-hydroxymethyl biphenyl derivatives, isoflavonoids, and 3,3-diphenylpentane derivatives [9].

## 9. Tissue Distribution

Numerous reports exist in the literature on the expression pattern of 5*α*-R1 and 5*α*-R2 in human tissue at various stages of development. The results vary due to differences in antibody sensitivity and specificity, mRNA analysis (in situ hybridization versus northern blotting versus reverse transcriptase-polymerase chain reaction), protein analysis (immunohistochemistry versus western blotting), tissue preparation, nature of tissue, evaluation of results, tissue fixation protocols, and control tissue. In addition, normal, benign, and malignant human tissue specimens are heterogeneous with variable expression of proteins among specimens from different individuals and within the same specimen, that is, inter- and intraindividual variability. Therefore, a summary of many studies that discussed the tissue distribution of 5*α*-R1-3 in different human tissues was tabulated to demonstrate differences in results (Table 3).

### 9.1. According to Age

#### 9.1.1. Fetus

Ellsworth and Harris [54] studied 5*α*-R activity in fetal scalp, back skin, and prostatic tissues and compared it to 5*α*-R activity in adult male scalp and prostatic tissues. They studied the conversion of radio-labeled T into DHT in relation to pH and response to selective 5*α*-R1 and 5*α*-R2 inhibitors and calculated the *k*_*m*_ of T at pH values of 7.0 and 5.5. 5*α*-R1 is expressed in fetal scalp and nongenital (back) skin at levels that are 5–50 times less than adult skin. 5*α*-R2 is expressed in the fetal prostate at levels similar to adult prostate. Thigpen et al. [13] studied 5*α*-R expression in fetal liver, adrenal, testis, ovary, brain, scalp, chest, and genital skin, using immunoblotting. They detected 5*α*-R2 only in fetal genital skin. Lunacek et al. [55] studied the expression of 5*α*-R 1 and 5*α*-R2 at the mRNA (RT-PCR) and protein (immunohistochemistry) levels in fetal and postnatal prostatic tissues until 6 years of age. Both 5*α*-R1 and 5*α*-R2 proteins were expressed in prostatic epithelial and stromal components, at consistent levels throughout all age groups. 5*α*-R1 is expressed mainly in the epithelium and 5*α*-R2 is expressed mainly in the stroma of the prostate. At the mRNA levels, both are detectable throughout the ages studied and both peak in the second trimester.

#### 9.1.2. Newborn-Onset of Puberty

In newborns, 5*α*-R1 is expressed at the protein level in the liver, skin, scalp [13] and prostate [55]. 5*α*-R2 is expressed in prostate, seminal vesicles, epididymis, liver, and to lesser extent in scalp and skin [13]. Hepatic expression of 5*α*-R1 and 2 is present at the protein level (immunoblotting) throughout postnatal life. At approximately 1.5 years, the expression of both proteins is not detected in the skin and scalp until the onset of puberty. At puberty, only 5*α*-R1 is reexpressed in the skin and scalp and persists thereafter until 81 years. In the prostate gland, Lunacek et al. reported that both 5*α*-R1 and 5*α*-R2 were detectable at the protein level using IHC until approximately 1 year of age. After that, they were detectable at the mRNA level (RT-PCR) until 6 years of age. Thigpen et al. only detected 5*α*-R2 at the protein level using immunoblotting in prostatic tissue from a 7-year-old male. Since the methods used by this group did not detect 5*α*-R1 protein in the newborn, juvenile, or adult prostatic tissues, and since other groups detected 5*α*-R1 at the protein level in fetal and adult benign prostatic tissue, 5*α*-R1 and 5*α*-R2 appear to be expressed in the prostate in male fetuses and throughout postnatal life.

#### 9.1.3. Adulthood-Old Age

5*α*-R1-3 is ubiquitously expressed [10, 11, 13, 20, 55]. 5*α*-R1 and 5*α*-R2 are expressed differently in liver, genital and nongenital skin, prostate, epididymis, seminal vesicle, testis, ovary, uterus, kidney, exocrine pancreas, and brain (Table 2, Aumuller et al.). Our laboratory described the expression of 5*α*-R3 using IHC in various benign and malignant tissues. 5*α*-R3 is overexpressed particularly in lung adenocarcinoma, testicular seminoma and yolk sac tumors, papillary thyroid cancer, and androgen-stimulated and castration-recurrent CaP relative to their benign counterparts [20]. When contrasting these data with the expressed sequence tag (EST) database from NCBI, both sets of data suggest a broad pattern of expression for 5*α*-R1-3 in human tissues; ESTs for 5*α*-R1 (271 sequences) have been reported from different human tissues, which include lung, brain, intestine, skin, prostate, testis, and stomach [56]. ESTs for 5*α*-R2 (39 sequences) have been reported in prostate, lung, liver, kidney, brain, testis, and skin [57]. ESTs for 5*α*-R3 (149 sequences) have been reported in kidney, testis, intestine, brain, liver, uterus, pancreas, skin, and prostate [58]. Tissue distribution of 5*α*-R3 protein in several human benign tissues was consistent with the tissue origin of the 5*α*-R3 EST reported at NCBI [20].

### 9.2. According to Organs

(See [10–13, 15–20, 54–61] (Table 3).)

## 10. Clinical Role of 5***α***-R

Alterations in the conversion of T into DHT by the enzyme 5*α*-R are associated with a number of human disorders:

### 10.1. 5*α*-R2 Deficiency (Pseudovaginal Perineoscrotal Hypospadias) [3, 62]

Studies in rabbit, rat, and human fetuses have shown that 5**α**-R activity is present in the urogenital sinus and external genital anlage prior to prostate and external genital differentiation. However, 5**α**-R activity is not present in the Wolffian duct, at the time of epididymal, vas deferens, and seminal vesicle differentiation. Thus, T and DHT have selective roles in male sexual differentiation during embryogenesis. T mediates Wolffian ductal differentiation, while DHT mediates male external genital and prostate differentiation.

5*α*-R2 deficiency is caused by decreased synthesis of DHT due to mutations in the 5*α*-reductase 2 gene. At least 50 mutations have been reported and is autosomal-recessive in the majority of patients. 5*α*-R2 deficiency results in a 46,XY disorder of sexual development (formerly male pseudohermaphroditism). Affected males are born with normal male internal reproductive structures (epididymis, seminal vesicles, and vasa deferentia); however, their external genitalia resemble those of females, that is, ambiguous genitalia. These individuals have a small penis that resembles an enlarged clitoris, labioscrotal fusion, and a urogenital sinus in which there are two separate urethral and vaginal openings. The vagina is short and blind ending. The testes are either in the labia, or inguinal canals or intra-abdominal. The vasa terminate at the blind-ending vaginal pouch. The prostate is hypoplastic, nonpalpable on rectal examination and is found to be rudimentary on transrectal ultrasound and MRI. Prostatic volumes are approximately (1/10)th of age-matched normal controls. Prostate biopsy reveals fibrous connective tissue, smooth muscle, and no identifiable epithelial tissue, which suggests atrophic epithelium or lack of epithelial differentiation. Plasma PSA is low or undetectable in these patients. Administration of DHT results in enlargement of the prostate. Neither BPH nor CaP has been reported in these patients. At puberty, these individuals undergo partial virilization of the external genitalia, although their secondary sexual hair remains sparse and they develop less male pattern baldness and acne despite normal sebum production. They undergo an increase in muscle mass, phallic growth, development of male body habitus, and deepening of the voice. Their libido is normal and they are capable of erections. Sperm production and fertility have been reported but depend on testicular location. The mechanism of partial virilization at puberty is through either the androgen receptor binding very high levels of serum T, albeit at lower affinity, or the increased expression of skin 5*α*-R1 at puberty, which results in peripheral synthesis of DHT from T or via the action of 5*α*-R3 through unknown mechanisms. 5*α*-R1 gene is normal in these subjects. No genetic deficiencies of the type 1 enzyme have yet been reported. Inactivation of the 5*α*-R1 gene in mice adversely affected reproduction in females. In addition, mice deficient in 5*α*-R1 manifest a parturition defect that is not reversed by the administration of DHT, but is reversed by 3*α*-androstanediol treatment. The biochemical features of this syndrome include the following.

- high normal to elevated levels of plasma T,
- low normal to decreased levels of plasma DHT,
- increased T to DHT ratio at baseline and following hCG stimulation,
- normal metabolic clearance of T and DHT,
- decreased levels of urinary 5**α**-reduced metabolites of C19 and C21 steroids, with increased 5*β*/5**α**urinary metabolite ratios,
- decreased plasma and urinary 3**α**-androstanediol glucuronide, a major metabolite of DHT,
- increased plasma levels of LH and/or FSH.

Phenotype, development and reproductive function in human females with 5*α*-R2 deficiency are unaffected.

### 10.2. LUTS Attributed to BPE

BPE results in a significant morbidity due to urethral obstruction and secondary detrusor dysfunction. Histological evidence of BPH is found in 50% of males by the age of 50 and 90% of males by the age of 80 [63]. The development of BPH depends on androgens, and BPH does not occur in men castrated prior to puberty [64]. 5*α*-R isozymes play significant roles in BPH development since DHT is the major androgen in the prostate. Patients with decreased DHT production due to 5*α*-R2 deficiency have a small prostate and BPH has not been reported [4]. In castrated dogs, treatment with either DHT or T results increased intraprostatic DHT and BPH [65]. However, coadministration of T with a 5*α*-R inhibitor decreased DHT formation and prevented BPH [66]. Finasteride and dutasteride have been shown to decrease circulating and intraprostatic DHT by 60–90%, and 90–98%, respectively [48–52]. Finasteride and dutasteride result in a decrease in prostate size by 20–25% through prostatic epithelial cell apoptosis and a significant improvement in LUTS. Furthermore, they change the natural history of the disease by decreasing the risk of acute urinary retention by 57–79% and decreasing BPH-related surgery by 48–69% [67, 68]. Although both isozymes are overexpressed in prostate tissues from patients with BPH [15], inhibition of 5*α*-R2 activity is the major contributor in the treatment of BPH as the additional inhibition of 5*α*-R1 activity by dutasteride does not appear to be of any further benefit in BPH treatment [69].

### 10.3. Primary Prevention of CaP [67, 68]

Both finasteride and dutasteride have been tested in large, prospective, randomized, placebo-controlled, double-blind studies as primary preventive therapies for CaP. The Prostate Cancer Prevention Trial (PCPT) randomized nearly 19,000 men at low risk for CaP into a treatment group, given finasteride at 5 mg/d and a control group, given placebo, who were followed up for 7 years. At the end of the study, participants were offered a prostate biopsy. For-cause biopsies were done for abnormal DRE and/or PSA > 4.0 ng/mL. PCPT showed that finasteride was effective at reducing the overall risk of biopsy detectable CaP by nearly 25% and this was due mainly to reduction in the risk of low-grade disease (Gleason sum <7). The reduction in the risk of CaP was seen across all subgroups, such as age, race, family history, and baseline PSA. Finasteride users also had improved BPH outcomes. The benefits of finasteride treatment occurred at the expense of a higher rate of diagnosis of moderate- and high-grade CaP (Gleason score ≥7) and more sexual adverse effects. The Reduction by Dutasteride of Prostate Cancer Events trial (REDUCE) studied the effect of dutasteride versus placebo in a large group of men at higher risk of CaP than in PCPT who had at least one negative prostate biopsy at baseline. The study lasted 4 years and participants received mandatory prostate biopsy at 2 and 4 years. Dutasteride decreased the risk of biopsy detectable CaP by nearly 24% and this reduction in risk was evident across all subgroups tested. The frequency of diagnosis of moderate- and high-grade CaP was unchanged over the entire length of the study and beneficial effects were observed on BPH outcomes. However, 12 Gleason score 8–10 cancers were detected in the dutasteride group at years 3-4 versus only one in the placebo group and dutasteride treatment was associated with more sexual adverse effects. The benefit of finasteride or dutasteride in reducing the risk of low-grade CaP is clear. Low-grade CaP is unlikely to be lethal and patients may reduce their risk of overtreatment. However, these drugs may induce high-grade CaP, a concern that has prevented FDA approval of finasteride and dutasteride use for CaP prevention. Several secondary analyses of these two trials have concluded that these drugs actually reduce the risk of moderate- and high-grade disease but these analyses are hypothesis-generating and not definitive. In March 2009, the American Urological Association and the American Cancer Society issued a “cautious” joint statement that accepted these drugs as an option to prevent CaP provided that they are used mainly after a thorough discussion of risks and benefits. In January 2011, the FDA's Oncologic Drug Advisory Committee voted against recommending either dutasteride or finasteride for the specific indication of CaP risk reduction because of the potential increased risk of high-grade disease.  Table 4 provides a comparison between PCPT and REDUCE.

### 10.4. Treatment of CaP

**(a) Biochemical Failure after Local Therapy with Curative Intent [70–73]**

Finasteride and dutasteride have been tried, singly and in combination, in patients with biochemical failure after radical prostatectomy or radiotherapy. The most common combination was a 5*α*-RI and a nonsteroidal antiandrogen. Finasteride and dutasteride monotherapy decreased serum PSA to variable extent. PSA decrease was more frequent and of greater magnitude in patients treated with an antiandrogen and 5*α*-RI versus 5*α*-RI alone. However, none of these trials studied the impact on disease-specific or overall survival and none compared 5*α*-RI mono- or combination therapy against 1st line androgen deprivation treatment in a randomized fashion.

**(b) CR-CaP**

CR-CaP was thought for many years to be androgen-independent or hormone-refractory but CR-CaP remains AR-dependent and probably AR-ligand dependent in almost all cases [74]. Despite castrate serum levels of T (<50 ng/dL), CR-CaP tissue levels of T and DHT were similar and 80–90% lower compared to their levels in benign prostatic tissue, respectively [75]. CR-CaP tissue synthesizes testicular androgens (T and DHT) in an intracrine fashion from several substrates such as cholesterol, progesterone, adrenal androgens, and androstandione [76–79]. Other phenomena intrinsically acquired by CR-CaP tissue in response to castration include the continuous expression of AR [80], upregulation of the synthesis of enzymes necessary for steroidogenesis [76], AR hypersensitivity (up to 10,000 times) to low levels of ligands by alteration of its co-activator profile from SRC1 to TIF2 and through its phosphorylation by SRC and Ack1 tyrosine kinases [81–83] and AR functional mutations, which broaden ligand specificity in 5–30% of cases [84].

New second-line hormonal therapeutic agents that have shown better performance in CR-CaP compared to the old generation of second-line hormonal therapies are abiraterone acetate and MDV3100, among others [77, 85]. Abiraterone acetate is a potent, selective, and irreversible inhibitor of CYP17A1 enzyme, which is an important enzyme in the intracrine synthesis of testicular androgens. MDV3100 inhibits ligand binding to the AR and nuclear translocation of AR-ligand complex. The clinical response to these new drugs is indirect proof that CR-CaP remains androgen stimulated. 5*α*-R isozymes are important in the growth of CR-CaP tissue since they are upregulated in CR-CaP and may contribute to intracrine synthesis of testicular androgens. These enzymes convert progesterone, ASD, and T into pregnanldione, androstandione, and DHT, respectively [74, 79]. Pregnanldione is further converted via several steps into androstandiol which is oxidized by 17*β*-hydroxysteroid dehydrogenase 2 and 10 (17*β*-HSD2 and 10) to DHT (the backdoor pathway to DHT synthesis). Androstandione is converted by 17*β*HSD3 into DHT.

Clinical trials of finasteride [70] and dutasteride [71] as monotherapy in patients with advanced CaP showed no improvement of clinical end points. The presence of 5*α*-R3 in CR-CaP is a potential explanation, until such time that an inhibitor has been proven effective clinically. Combination therapy of 5*α*-RI with antiandrogen or ketoconazole and hydrocortisone was tried in CR-CaP as second or third line hormonal therapies [86–89]. PSA decreases of variable magnitudes and durations were achieved in more than half the patients. However, none of the combination trials were designed to test the effect on disease-specific and overall survival. In a phase II, single arm study of 57 patients with CR-CaP, dutasteride added to ketoconazole, and hydrocortisone reduced PSA ≥ 50% of baseline value in 56% of patients, responses that lasted for a median of 20 months. Median time to disease progression was 14.5 months that was better than all prior studies of ketoconazole and hydrocortisone in CR-CaP [89].

### 10.5. Androgen-Stimulated Skin Disorders (Acne, Androgenic Alopecia and Hirsutism)

Hyperandrogenism, or excessive androgen production, is primarily a disorder of females. Polycystic ovary syndrome (PCOS) is the most common cause of female hyperandrogenism with a prevalence of 6% to 10% in women of childbearing age [90]. Other causes of hyperandrogenism include androgen-secreting ovarian and adrenal tumors, congenital adrenal hyperplasia, and exogenous androgenic hormone administration. Regardless of the cause, overproduction of either T or T precursors leads to exaggerated T action in target tissues such as skin. In skin, T is converted to DHT by the enzyme 5*α*-R, which acts directly on hair follicles and the sebaceous glands. The most frequent dermatologic manifestations of androgen excess are hirsutism, acne, and androgenic alopecia [91].

Androgen surge can miniaturize hair follicles resulting in male androgenic alopecia at puberty and in the scalp of genetically predisposed individuals. In women, androgen excess plays a role in scalp hair loss; however, this process is different than in men and is referred to as female pattern hair loss. Hirsutism is a disorder of excessive growth of terminal hair in women, in a male-like distribution, which is stimulated by androgen excess [92]. In acne, the pubertal androgen surge increases the stimulation of sebaceous glands resulting in increased sebum production and acne formation in susceptible individuals [93].

In all androgen-stimulated skin disorders, the activity of 5*α*-R enzyme system is increased such as in the hair follicles of hirsute women [93], in balding scalps [94], and in acne-prone skin [95]. Inhibition of the 5*α*-R enzyme system appears to be a target for treatment of androgen-stimulated skin disorders, since 5*α*-R inhibitors may result in fewer side-effects by not blocking the action of T, unlike classical anti-androgens such as cyproterone acetate or spironolactone.

Finasteride and dutasteride reduce scalp DHT levels by 64% and 51%, respectively [96, 97]. In men with androgenic alopecia, finasteride and dutasteride significantly increased hair count after a minimum of 6-month treatment [98, 99]. While finasteride 1 mg daily was superior to 5% topical minoxidil in inducing hair growth [100], finasteride 5 mg daily was inferior to dutasteride 2.5 mg daily in a phase II study as treatment for male androgenic alopecia [97]. Finasteride and topical minoxidil (but not dutasteride) are FDA-approved for male androgenic alopecia. In women with androgenic alopecia, neither finasteride nor dutasteride is FDA-approved treatment options due to teratogenicity. Finasteride has been tested in postmenopausal women at the 1 mg dose without success. However, finasteride was shown to be effective in 4 women with elevated serum T levels [101]. Whether finasteride is only effective in women with hair loss and hyperandrogenism or that higher doses are needed in women remains to be tested. A single case report showed improvement with dutasteride in a woman who had failed to respond to finasteride [102].

5*α*-RI also is acceptable therapy for hirsutism. Finasteride 5 mg/day was superior to placebo and similar to spironolactone and flutamide in reducing the severity of hirsutism [103]; however, in a different study, finasteride was inferior to flutamide as a treatment for hirsutism [104]. Dutasteride has not been tested as a treatment for hirsutism.

The role of 5*α*-RI is unclear for treatment of acne. MK-386, a selective 5*α*-R1 inhibitor, decreased sebum DHT levels in a dose-dependent fashion [105]. MK-386 was examined as a treatment for acne in a placebo-controlled trial and was found to be similar to placebo and inferior to systemic minocycline therapy. Furthermore, MK-386 did not enhance the therapeutic benefit of minocycline when used in combination [106]. In another study, finasteride decreased the severity of acne but was inferior to flutamide and cyproterone acetate with ethinyl estradiol [107]. No studies of dutasteride for treatment of acne have been reported.

## 11. Conclusions

The 5*α*-R system is an important player in human physiology and pathology. More work is needed to identify the biochemical characteristics and role of 5*α*-R3 in several human conditions such as CaP and androgen-stimulated skin diseases. Clinical data are inconclusive regarding the benefit of 5*α*-RI for CaP prevention. Clinical trials are ongoing to define the role of dutasteride for treatment of CaP, such as REDEEM (dutasteride in low-risk CaP patients on active surveillance), ARTS (biochemical failure after local treatment with curative intent), AVO 108943 (bicalutamide and dutasteride versus bicalutamide and placebo in CR-CaP), and ARI40006 (2-year follow-up study of REDUCE participants who received dutasteride or placebo) [108]. Future trials should focus on blocking multiple steroidogenic enzymes at once, such as in men with biochemical failure after local therapy or men with CR-CaP. Blocking several different steps in steroidogenesis simultaneously may not allow CaP cells time to adjust to loss of androgen stimulation.

## Figures and Tables

**Table 1:** Different steroid families.

Class	Example	Number of carbon atoms
Steroid backbone	Gonane	17
Estranes	Estradiol	18
Androstanes	Testosterone	19
Pregnanes	Progesterone	21
Glucocorticoids	Cortisol	21
Mineralocorticoids	Aldosterone	21
Cholanes	Cholic acid	24
Cholestanes	Cholesterol	27

**Table 2:** Comparison between finasteride and dutasteride (referenced in text).

	Finasteride	Dutasteride
Family	Steroidal 5*α*-RI (4-azasteroid)	Steroidal 5*α*-RI (4-azasteroid)

IC_50_ for 5*α*-R1, 2 and 3 (nM)	360, 69, 17.4	7, 6, 0.33

FDA-approved clinical uses	Male androgenic alopecia Benign prostatic enlargement	Benign prostatic enlargement

Clinical dose	1 mg daily for male androgenic alopecia 5 mg daily for benign prostatic enlargement	0.5 mg daily

Half-life (T 1/2)	6–8 hours	5 weeks

Suppression of DHT	↓Serum DHT by 71%	↓Serum DHT by 95%

↓Intraprostatic DHT by 85%	↓Intraprostatic DHT by 97–99%

**Table 3:** Tissue distribution of 5*α*-reductase 1–3 according to different authors.

Author isozymes studied	Tissue type	Materials and methods	5*α*-R1	5*α*-R2	Notes
Eicheler et al. [10] 5*α*-R1-2	Epidermis: genital (scrotum) nongenital (Axilla, breast, lip, eyelid) using a semiquantitative visual scale	Protein expression (IHC) using rabbit polyclonal antibodies against synthetic peptides from C-terminus parts of 5*α*-R1-2 proteins. Antibody sensitivity and specificity confirmed by ELISA and WB on FFPE biopsy or autopsy tissues	Nuclear, in epidermis from all sites: (scrotal> axilla> breast> lip> eye lid): stratum basale (++), stratum spinosum (++), absent in stratum granulosum and stratum corneum, dermal papillae, fibrous and outer epithelial RS (++), inner epithelial RS (+), matrix cells of hair bulb (++), scrotal fibroblast (++), basal and secretory cells of sebaceous glands (++), secretory and myoepithelial cells of sweat glands (++), arrector pili muscles (+), dermal adipocytes (+). No qualitative differences in males and females	Cytoplasmic, in epidermis from all sites: stratum spinosum (++), stratum basale (+), absent in stratum granulosum and stratum corneum, inner epithelial RS (++), matrix cells of hair bulb (+), absent in dermal papillae, fibrous and outer epithelial RS, basal (+) and glandular (−) cells of sebaceous glands, myoepithelial (+) and secretory cells (−) of sweat glands, dermal adipocytes (+) No qualitative differences in males and females	5*α*-R1 more uniformly spread in skin versus 5*α*-R2 that is mainly found in inner epith RS

Aumüller et al. [11] 5*α*-R1-2	Many tissue types, using a semiquantitative visual scale	Protein expression (IHC) using rabbit polyclonal antibodies against synthetic peptides from C-terminus parts of 5*α*-R1-2 proteins. Antibody sensitivity and specificity confirmed by ELISA and WB. Tissues from surgical pts and autopsies, fixed in Bouin's or formaldehyde	Mainly nuclear: Prostate: stroma (+), epithelium (+) Seminal vesicle: stroma (+), epithelium (+) Epididymis: stroma (+), epithelium (+) Testis: Leydig cells (+), Sertoli cells (+) Ovary: stroma (++), theca and granulosa cells (−) Uterus: endometrium (+), myometrium (+) Liver: hepatocytes (+/−), bile duct c (+), kupffer cells (++) Pancreas: exocrine (+), islets of Langerhans (−) Kidney: glomerulus (+), PT (−), DT (++), CD (+) Adrenal: cortex (−), medulla (−) Thyroid: thyrocytes (−), C cells (−) Cerebral cortex: pyramidal c (+), glial cells (+/−) Pituitary: prolactin cells (+), others (−)	Mainly cytoplasmic: Prostate: stroma (+), epithelium (++), specially basal cells Seminal vesicle.: stroma (+), epithelium (++) Epididymis: stroma (+), epithelium (+) Testis: spermatogonia (+), Leydig and Sertoli cell (−) Ovary: stroma (+), theca and granulosa cells (+/−) Uterus: endometrium (+), myometrium (+) Liver: hepatocytes (++), bile duct c (+), kupffer cells (−) Pancreas: exocrine (+), islets of Langerhans (−) Kidney: glomerulus (−), PT (++), DT (+/−), CD (+) Adrenal: ZG (+), ZF (+/−), ZR (+/−), med (−) Thyroid: thyrocytes (−), C cells (−) Cerebral cortex: pyramidal c (++), glial c (−) Pituitary: prolactin cells (+), others (−)	5*α*1-2 is ubiquitous

Bayne et al. [12] 5*α*-R1-2	Scalp biopsies from bald and non-bald men	Protein expression (IHC) using validated mouse monoclonal antibodies against peptides from N-terminus parts of 5*α*-R1-2 and enzyme activity using ^3^H-T	In balding and non-balding scalp: 5*α*-R1 is expressed only in sebaceous glands No expression was detected in hair follicles or in epidermis	In balding and nonbalding scalp: 5*α*-R2 is expressed in infundibula, outer (mainly) and inner epithelial RS of hair follicles. No expression was detected in dermal papillae or in sebaceous glands	

Thigpen et al. [13] 5*α*-R1-2	Autopsy and surgical tissue samples	Messenger RNA (NB) and protein expression (IHC and WB) using rabbit polyclonal antibodies against peptides from C-terminus parts of 5*α*-R1-2	5*α*-R1 protein is expressed in liver and chest skin and 5*α*-R1 mRNA was detected in cerebellum, hypothalamus, pons, medulla oblongata, skin, and liver 5*α*-R1 was not detected by WB in any fetal tissue. 5*α*-R1 was detected by WB in newborn liver, skin, and scalp.5*α*-R1 was detected by WB in all scalp samples from balding and nonbalding men (except one). It was not detected in any normal prostate, BPH, or PC sample	5*α*-R2 protein is expressed in prostate, seminal vesicles, epididymis, and liver. 5*α*-R2 mRNA was detected in prostate, SV, epididymis. and liver. 5*α*-R2 was detected by WB only in fetal genital skin (not detected in fetal liver, adrenal, testis, ovary, scalp, and brain). 5*α*-R2 was detected by WB in newborn prostate, SV, epididymis, liver, skin. and scalp. 5*α*-R2 was not detected by WB in any sample of balding and nonbalding scalp from one man. It was detected in all normal prostate, BPH, and PC samples	5*α*-R1 protein was not detected in any prostate sample

Thomas et al. [14] 5*α*-R1-2	Prostate: BPH (TURP), ASCaP (RP), CaP metastasis in androgen deprivation-treated men (autopsy)	Protein expression (IHC) using rabbit polyclonal antibodies against peptides from N-terminus of 5*α*-R1-2 proteins. Antibody specificity confirmed with WB on naïve, 5*α*-R1- and 5*α*-R2- transfected COS-1 cells and IHC on transfected COS-1 cells-Evaluated by measuring percentage of moderate- and high-intensity immunostaining areas in relation to total epithelial, PIN, or tumor area in PE tissues	Nuclear in BPH, shifts to cytoplasm in HGPIN and CaP Immunostaining intensity: Metastasis> CR-CaP> AS-CaP = HGPIN> BPH	Mainly cytoplasmic in all specimens Immunostaining intensity: BPH = Metastasis = CR-CaP> AS-CaP = HGPIN	All differences are statistically significant

Thomas et al. [14] 5*α*-R1-2	Prostate: AS-CaP (RP) with Gleason score <7, 7, >7	Protein expression (IHC) using validated rabbit polyclonal antibodies, evaluated by visually estimating percent of total tumor area showing low-, moderate, and high-intensity in relation to Gleason score	Mainly nuclear in BPH, nuclear and cytoplasmic in CaP (all grades), and in adjacent benign epithelial tissue Immunostaining intensity: High grade > moderate grade = low grade > PC-adjacent benign tissue > BPH	Mainly cytoplasmic in all samples (benign and malignant) Immunostaining intensity: BPH> high grade> moderate grade = low grade = adjacent benign tissue	Staining in CaP-adjacent benign tissue is not significanty different from low- and high-grade CaP for either isozyme

Söderstöm et al. [15] 5*α*-R1-2	Prostate: BPH (TURP) and AS-CaP via RP or RC	Messenger RNA expression (sqRT-PCR) and measurement of 5*α*-R enzyme activity at pH 5.5 and 7 using ^14^C-T at 37°C in homogenized frozen pulverized prostate tissue	5*α*-R1 mRNA expression is similar in BPH and AS-CaP. There was no correlation between enzyme activity at pH (5.5 and 7) and 5*α*-R1 mRNA expression as expressed on the basis of *β*-actin	5*α*-R2 mRNA and enzyme activity were higher in BPH than in AS-CaP. There was a positive correlation between enzyme activity at pH 5.5 and expression of 5*α*-R2 mRNA as expressed on the basis of *β*-actin	

Lehle et al. [61] 5*α*-R 1-2	BPH and CaP tissue post prostate biopsy or RP frozen in liquid nitrogen, one human liver sample	Messenger RNA expression (ISH, sqRT-PCR)	ISH showed that 5*α*-R1 mRNA is expressed in epithelium > stroma mRNA expression levels by sqRT-PCR: liver> CaP > BPH> Normal prostate	ISH showed that 5*α*-R2 mRNA is expressed in epithelium > stroma mRNA expression levels by sqRT-PCR: liver = BPH > Normal prostate > CaP	

Habib et al. [16] 5*α*-R1-2	BPH tissue (TURP) frozen in liquid nitrogen or in ice-cold RPMI with FBS and archival PE-BPH tissue	Messenger RNA expression (ISH, RT-PCR) and measurement of enzyme activity at pH 5 and 7.5 using ^3^H-T at 37°C in homogenized pulverized frozen prostate tissue	5*α*-R1 mRNA expressed in epithelium > stroma	5*α*-R1 mRNA expressed in epithelium > stroma 5*α*-R2 mRNA > 5*α*-R1 mRNA in BPH 5*α*-R2 enzyme activity ≫ 5*α*-R1 enzyme activity in homogenized BPH tissue	

Bonkhoff et al. [17] 5*α*-R1-2	BPH (TURP), AS-CaP (RP), CR-CaP (channeling TUR)	Protein expression (IHC) using polyclonal rabbit antibodies against peptides from C-terminus parts of 5*α*-R1 and 2, validated by ELISA and WB	Mainly nuclear, in normal prostate and BPH tissue (luminal epithelial > basal) and in stroma 5*α*-R1 immunostaining > 5*α*-R2 in BPH (in both epithelium and stroma). In CaP, 5*α*-R1 immunostaining became nuclear and cytoplasmic and more intense in HGPIN and CaP versus adjacent benign tissue (specially CR-CaP)	Mainly cytoplasmic (weak), in normal prostate and BPH tissue (basal > luminal epithelial) and stroma. In CaP, 5*α*-R2 immunostaining became nuclear and cytoplasmic and more intense in HGPIN and CaP versus adjacent benign tissue (specially CR-CaP)	

Shirakawa et al. [18] 5*α*-R1-2	BPH (RC) fixed in formaldehyde and paraffin-embedded	Messenger RNA (qRT-PCR), protein (IHC) expression using polyclonal rabbit antibodies against peptides from C-terminus parts of 5*α*-R1 and 2, validated by ELISA and enzyme activities at pH 5 and 7.5 using ^3^H-T at 37°C	5*α*-R1 mRNA copy numbers> 5*α*-R2 mRNA in BPH 5*α*-R1 protein expression is intense in epithelium of BPH (higher than 5*α*-R2 protein) 5*α*-R1 enzyme activity at pH 7.5 is similar to 5*α*-R2 enzyme activity at pH 5.0	5*α*-R2 mRNA < 5*α*-R1 mRNA in BPH 5*α*-R2 protein expression is detected in epithelium and stroma of BPH (less intense than 5*α*-R1)	

Titus et al. [19] 5*α*-R1-2	ASBP, AS-CaP and CR-CaP (RP or channeling TURP) tissue that was FFPE or snap frozen in liquid nitrogen	Protein expression (by IHC in TMAs that are quantified by visual scoring and digital image analysis and by WB) and enzyme activity in homogenized pulverized prostate tissue using ^3^H-ASD at 37°C	5*α*-R1 is nuclear and cytoplasmic in all 3 tissues Nuclear 5*α*-R1 staining intensity: ASBP = AS-CaP = CR-CaP Cytoplasmic 5*α*-R1 staining intensity: ASBP **= **AS-CaP> CR-CaP Not detected in stroma in any of the 3 tissues In WB, 5*α*-R1 > 5*α*-R2 in all 3 tissues 5*α*-R1 enzyme activity > 5*α*-R2 in CR-CaP (3 folds)	5*α*-R2 is mainly cytoplasmic in all 3 tissues Nuclear 5*α*-R2 staining intensity: ASBP = AS-CaP> CR-CaP Cytoplasmic 5*α*-R2 staining intensity: ASBP = AS-CaP> CR-CaP Not detected in stroma in any of the 3 tissues In WB, 5*α*-R2 was undetectable in CR-CaP 5*α*-R2 activity > 5*α*-R1 in ASBP and AS-CaP	In all 3 tissues, expression of 5*α*-R1 is consistenty more than 5*α*-R2 (in nucleus) but similar in cytoplasm

Godoy et al. [20] 5*α*-R3	Benign and malignant human tissue TMAs	Protein expression (IHC) using validated monoclonal mouse antibody against peptide from N-terminus of 5*α*-R3 protein and quantified by visual scoring and digital image analysis	5*α*-R3 was mainly cytoplasmic Benign tissue immunostaining: Kidney (PT,DT ++), liver (++), exocrine pancreas (++), skeletal muscle (+), skin (strata basale and spinosum ++), gastric epithelial cells (+), myometrium (++) Malignant tissue: colon adenoCA (++), esophageal adenoCA (++), RCC (++), HCC (++), ovarian mucinous CA (++), stomach adenoCA (++), testis seminoma and YS tumor (++), thyroid papillary CA (++), endometrioid CA (++), breast CA (+) ASBP: basal epithelial cells, HGPIN: benign basal and neoplastic epithelial cells, AS-CaP and CR-CaP: in neoplastic cells (5*α*-R3 immunostaining intensity: AS-CaP = CR-CaP > ASBP)		5*α*-R3 protein expression ↑ in the cytoplasm of malignant cells versus benign cells in prostate, testis, thyroid, lung and breast CA

Yamana et al. [21] 5*α*-R3	20 benign human tissues, CaP, and breast cancer cell lines	5*α*-R1-3 mRNA expression (RT-PCR) and measurement of 5*α*-R 1–3 enzyme activity using ^14^C-labelled ASD and T in intact cells in culture	5*α*-R3 expression at the mRNA level is higher than 5*α*-R1 and 2 in frontal cortex, heart, colon, stomach, liver, pancreas, lung, BPH, prostate, testis, mammary gland, brain, cervix, ovary, dermis, epidermis, total skin, small intestine, spleen, and kidney 5*α*-R2 mRNA was the most abundant in BPH and muscle Finasteride inhibits 5*α*-R2 and 5*α*-R3 with similar potency (IC_50_ = 14.3 nM, 17.4 nM, resp.). Dutasteride is a more potent inhibitor of 5*α*-R3 than finasteride (IC_50_ = 0.33 nM)		5*α*-R3 is ubiquitous Dutasteride is a triple 5*α*-RI *in vitro *

ELISA: enzyme-linked immunosorbant assay; IHC: immunohistochemistry; WB: western blot; NB: northern blot; FFPE: formalin fixed paraffin embedded; RS: root sheath; RP: radical prostatectomy; sqRT-PCR: semiquantitative reverse transcriptase-polymerase chain reaction; PE: paraffin-embedded; RPMI: Roswell Park Memorial Institute; FBS: fetal bovine serum; PT: proximal tubules; DT: distal tubules; CD: collecting ducts; TURP: transurethral resection of prostate; RC: radical cystectomy; HGPIN: high-grade prostate intraepithelial neoplasia; ISH: *in situ* hybridization; RCC: renal cell carcinoma; HCC: hepatocellular carcinoma; adenoCA: adenocarcinoma; CA: carcinoma; YS: yolk sac; TMA: tissue microarray.

**Table 4:** Comparison between PCPT and REDUCE.

	PCPT	REDUCE
Sponsor	South West Oncology Group	GalxoSmithKline
Duration	7 years	4 years
Risk of CaP in participants	lower	higher
No. of participants	18,882 randomized 9060 included in final analysis	8122 randomized
Age	≥55 years	50–75 years
Entry serum PSA	≤3.0 ng/mL	2.5–10 ng/mL
Baseline biopsies	No	Yes (6–12 cores) within 6 months prior to enrollment
Study-mandated biopsies	Year 7	Years 2 and 4
Study-mandated biopsy cores	≥6 (6 cores in nearly 80%)	10 (83% had at least 1 biopsy)
Overall relative risk reduction in CaP versus placebo	25%	23%
Incidence of Gleason sum ≥7 CaP	↑26% (6.4% in finasteride versus 5.1% in placebo), *P* < 0.05	Same (6.7% in dutasteride versus 6.8%: in placebo)
Incidence of Gleason sum ≥8 CaP	↑91% (2.1% in finasteride versus 1.1% in placebo), *P* < 0.05	Same over 4 years (0.9% in dutasteride versus 0.6% in placebo); however, in years 3-4, there were 12 GS ≥8 CaP in dutasteride group (0.5%) versus 1 in placebo group (<0.1%), *P* < 0.05
